# Ethical considerations for genetic research in low-income countries: perceptions of informed consent, data sharing, and expectations in Nicaragua

**DOI:** 10.1038/s41431-023-01505-7

**Published:** 2023-12-05

**Authors:** Iris S. Delgado, Abigail Outterson, Vaishnavi Ramesh, Alda Gabriela Amador Sanchez, Alfonso César Boza, Damaris Lopez-Pilarte, Juan José Amador Velázquez, David J. Friedman, Daniel R. Brooks, Madeleine K. Scammell, Catharine Wang

**Affiliations:** 1 Department of Epidemiology, Boston University School of Public Health, Boston, MA USA; 2 Department of Environmental Health, Boston University School of Public Health, Boston, MA USA; 3 Renal Division, Beth Israel Deaconess Medical Center, Boston, MA USA; 4 Department of Community Health Sciences, Boston University School of Public Health, Boston, MA USA

**Keywords:** Ethics, Genomics

## Abstract

Genetic research presents numerous ethical, legal, and social implications (ELSI), particularly when the research involves collaborations between investigators in high and low-income countries. Some ELSI issues are universal, and others are specific to context and culture. This study investigates perceptions of genetic research in Nicaragua, Central America, where local and U.S. based researchers have collaborated for over a decade. A total of 43 residents from northwestern Nicaragua, a region with high mortality rates attributed to chronic kidney disease of non-traditional causes (CKDnt), were interviewed, including research participants in ongoing studies (*n* = 36), health professionals (*n* = 3), labor leaders (*n* = 2), and family members of research participants (*n* = 2). Questions focused on informed consent, data-sharing, and post-study expectations. Audio recordings of interviews conducted in Spanish were transcribed and translated into English. English transcripts were coded and analyzed using NVivo 12 software. The lack of familiarity with terms in the consent form presented a barrier to participant comprehension of key elements of the genetic research study, raising concerns about the validity of informed consent. Research participants often viewed their participation as access to health care. Health professionals emphasized the importance of long-term partnerships between foreign-based researchers and local health institutions. Leaders and family members recommended that they be informed of research studies and allowed the opportunity to consent, as they felt the benefits and risks of research also apply to them. Our findings identified genetic research practices to be improved upon in order to be more responsive to the contextual realities of collaborators living in low-resource settings.

## Background

Since the successful mapping of the human genome, there has been increasing interest in the potential of genomics to identify genes associated with a higher risk of disease onset [1]. Genome-wide association studies (GWAS) have detected genetic contributions to complex diseases that also have environmental risk factors, such as heart disease, diabetes, and chronic kidney disease [2–4]. There are, however, many challenges in interpreting and generalizing these associations [5, 6]. Most significant is that the majority of GWAS have primarily involved participants of European descent [7]. Population groups vary in ancestry, exposures, and surroundings, making it unlikely to adequately predict risk and develop proper interventions without the participation of diverse communities in genetic research [8]. Associations may not be applicable to populations not well represented in genetic databases, such as those in low and middle-income countries (LMICs) with the greatest burden of chronic disease [9].

Studies mainly funded by the U.S. and European governments are attempting to reduce this disparity in genomic data by soliciting the participation of under-represented communities in LMICs [10]; most of whom experience inequities in resources, power, health, and information, and may be unfamiliar with genetic research terms, methods, technologies, and implications [11]. Previous studies that have explored stakeholder perspectives of genetic research in LMICs and Indigenous communities found that obtaining valid informed consent, where research information is provided in an understandable way, is a substantial ethical dilemma [12–16]. Additional ethical, legal, and social implications (ELSI) also arise related to perceptions of data ownership, use, and sharing policies; privacy and confidentiality concerns; therapeutic misconceptions; and the effect of findings on families and communities [17–19].

Little to no studies have sought to understand stakeholder perspectives of genetic research in Central America. This qualitative study aimed to understand ethical challenges in the conduct of research that uses GWAS to identify genetic variants that may contribute to kidney disease risk in northwestern Nicaragua.

### CKDnt research in Nicaragua

Chronic kidney disease (CKD), the gradual failure of kidney function, contributes a significant burden to global health [20]. Traditional risk factors for CKD include advanced age, diabetes, and high blood pressure [21]; however, a form of chronic kidney disease due to non-traditional causes (CKDnt) has been impacting rural communities throughout Central America [22, 23].

For over a decade, The Boston University Research Group for the Study of Chronic Kidney Disease in Central America has conducted studies along the Pacific coast of Nicaragua, where CKDnt is a leading cause of mortality [24, 25]. Results of studies by our group and others suggest that the occurrence of CKDnt is highest among certain occupational groups, such as sugarcane workers, brickmakers, and miners [26, 27]. Although much of this research has focused on environmental and occupational contributors, we are also examining the role of genetic susceptibility in CKDnt causation [28, 29].

To date, we have enrolled over 1500 current and former workers in regions with high CKDnt burden in northwestern Nicaragua as participants in an ongoing genetic case-control study, with initial funding from private and foundation sources and current funding from the National Institutes of Diabetes and Digestive and Kidney Diseases (NIH-NIDDK). Participants consented to the collection and sharing of genetic data within publicly accessible databases, i.e., dbGAP [30, 31] as well as the return of urine dipstick, blood chemistry/electrolytes, and hematology results (but not genetic results).

This embedded study investigated research participant and community stakeholder perceptions of ongoing genetic research, including their understanding of genetic risk and its relevance in disease causation, thoughts on the informed consent process and policies, and expectations of research.

## Methods

### Study sites and participants

Participants of this qualitative study represent three departments and five municipalities in the Pacific coastal regions of Nicaragua where genetic study enrollment took place from October 2018 to February 2021. In addition to participants in the genetic study who underwent the original consent process, we included participants in a separate National Institute of Environmental Health Sciences (NIH-NIEHS) funded cohort study conducted in the same region who, more than a year after consenting for the initial study, underwent a brief supplemental consent process specific to the sharing of their genetic data. We included this group because we were interested in ethical concerns that may have arisen or been alleviated by a more recent separate and condensed consenting process. In all instances, the entire consent form was read to each participant, which took an average of 15 minutes for the original NIDDK consent and about 5 minutes for the supplemental NIEHS consent.

We also included participants’ family members, health workers, and leaders in communities with a high prevalence of CKDnt to learn their reactions to the different consent processes and U.S. data-sharing policies. We explained two types of consent to community members; where broad consent allows the use of data and samples in future research of any kind, while specific consent allows the use of data and samples in immediate, not future, research [32].

### Ethics approval and consent to participate

The qualitative study protocol was approved by the Boston University Medical Campus Institutional Review Board (BUMC IRB). Protocol (H-40933) underwent an exempt limited review process as an administrative supplement to the NIH-NIDDK parent grant (H-32414), approved by the BUMC IRB and Ethics Committee in Nicaragua (CIRE). All participants gave consent before the interview.

### Study procedures

We contacted potential participants by phone using a purposive sampling approach. We described the aims of the qualitative study, specific activities, the voluntary nature of participation, and the confidentiality of the information. In-person semi-structured interviews were administered in Spanish by local researchers (AGAS and ACB) over six consecutive weeks, from April to June 2021. Interview guides were tailored for each stakeholder group. Local researchers took turns leading each interview and taking observational notes. Following the interview, the researchers shared an infographic depicting the genetic study research aims (see Appendix A). Each participant received a stipend of 200 Córdobas (~$6 USD).

**Fig. 1:**
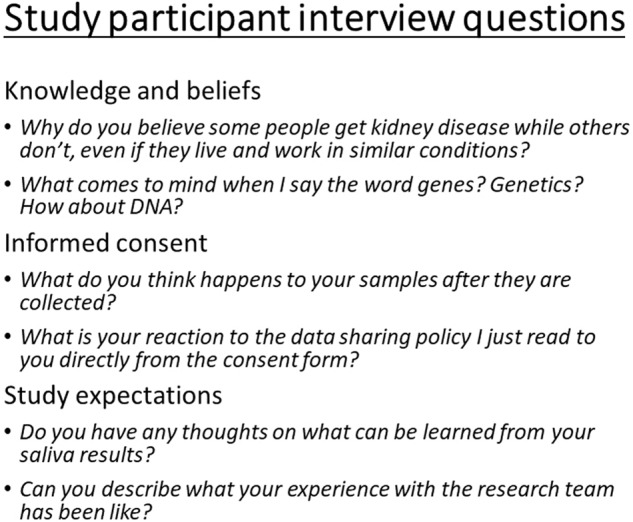
Study participant interview questions. A few examples of questions asked to genetic study participants by topic of interest.

The study protocols and interview guides were developed with the local research staff. We conducted preliminary interviews using a continuous improvement evaluation model and gathered participant and interviewer feedback on the flow and phrasing of questions that would lead to the collection of intended data. Some examples of interview questions are shown in Fig. 1.

We also sought to determine how easy or difficult the parent study consent forms were to comprehend, so we assessed for readability using an adaptation of the SMOG (Simple Measure of Gobbledygook) index [33]. The SOL scale, also referred to as the Spanish SMOG, indicates the years of education a participant would need to comprehend a written document in Spanish by counting the number of polysyllabic words and sentence lengths in a document [34, 35] (See Appendix B).

### Data analysis

Spanish audio interviews were transcribed by local researchers AGAS and ACB, and later de-identified and translated into English by ISD. English transcripts were imported into qualitative data analysis software NVivo 12 to code and analyze for themes in understanding terms, concepts, data sharing policies, and aims of the research, as stated in the informed consent. VR, AO, and ISD read the transcripts and each developed an initial codebook using a data-driven inductive approach [36]. The codebooks were compared and a deductive framework was developed and applied to limit themes to our research questions. Multiple versions were developed by VR, AO, and ISD and finalized in discussion with other members of the research team.

## Results

### Participant demographics

A total of 43 individuals participated in this qualitative study: 36 research study participants and seven community stakeholders, representing municipalities in Chinandega (33%), León (53%), and Managua (14%). Of the 36 research participants, 20 were enrolled in the NIDDK genetics study, and 16 were originally enrolled as NIEHS cohort study participants and later consented to GWAS data sharing. All research participants were male, as this was the inclusion criteria for enrollment into both parent studies. The average research participant age was 37, ranging from 22–58 years of age. About 60% of research participants had at most a primary school level of educational attainment. Of the seven community stakeholders, three were health professionals, two were labor union leaders, and two were family members of genetic study participants. These and other demographics are listed in Table 1.

**Table 1:** Study participant demographics.

	NIDDK genetic study	NIEHS cohort study	Community stakeholder
*n* (%)	20 (47)	16 (37)	7 (16)
Sex			
Male	20 (100)	16 (100)	4 (57)
Female	-	-	3 (43)
Region			
Chinandega	6 (30)	5 (31)	3 (43)
León	14 (70)	5 (31)	4 (57)
Managua	-	6 (38)	-
Industry			
Sugarcane	8 (40)	11 (69)	1 (14)
Brick	4 (20)	4 (25)	-
Mining	8 (40)	-	1 (14)
Plantain	-	1 (6)	-
Healthcare	-	-	3 (43)
Commerce	-	-	2 (29)
CKDnt status			
Yes	10 (50)	-	-
No	10 (50)	-	-
Health insurance			
Yes	16 (80)	10 (62)	-
No	4 (20)	6 (38)	-
Highest education			
No formal education	1 (5)	1 (6)	-
Learned to read	6 (30)	-	-
Primary school	7 (35)	5 (31)	-
Secondary school	-	7 (44)	-
Trade school	3 (15)	-	-
University	3 (15)	3 (19)	-
Age (mean (sd))	42 (7.7)	30 (7.9)	-

### Ethical issues by domains of genetic research

Interviews highlight specific ethical issues related to the following domains of conducting genetic research: 1) knowledge and beliefs about the genetic etiology of disease, 2) informed consent, 3) data use and sharing policies, 4) benefits and risks of participation, and 5) post-study expectations. Within each domain, themes are presented along with opinions and views that differed among research participants from the two parent studies and community members.

### Knowledge and beliefs about the genetic etiology of CKDnt

Participants understood basic concepts of genes and disease through their lived experiences. Nearly all study participants (94%) acknowledged associations of hereditary factors to health and disease by naming diseases that affect more than one family member; most listed were kidney disease, diabetes, heart disease, hypertension, and/or various cancers.

Two research participants expressed a deterministic and inevitable view of disease. Genetic-related causes of disease were referenced as being born with strength or insufficiency in the genes, and compared to a contagious virus:

*“Genes and health go together like the links of a chain. That is where one inherits all the strength or no strength to a determined disease. One can acquire a mild flu, and another definitely does not acquire it, is immune.” (Miner, NIDDK)*

*“We have a bad gene in the body, so the kidneys do not work well. The bad gene causes the liquids that we normally expel through our body, to stay in the body and accumulate. Those are bad genes.” (Former sugarcane worker, NIDDK)*

At least five research participants noted that CKDnt has come to affect recent generations, and was not experienced in their grandparents’ generation. They were also more likely to emphasize that exposures to environmental factors like excess sun and heat, contaminated water, and air due to nearby industries are significant causes of CKDnt.

*“I think that, as it is today, it is the factories that intoxicate the air we breathe and discard waste liquids that end up in the rivers and the potable water. We do not drink it.” (Sugarcane worker, NIEHS)*

The majority of research participants (78%) believed kidney disease was also caused by individual behaviors and environmental conditions, and not determined solely by genetics. Poor individual behaviors and harmful personal habits, like not drinking enough water, not wearing adequate protective gear when applying chemicals, overexertion at work, excess sun exposure, alcohol use, and poor nutrition, were often cited as at least partial causes of CKDnt. A combination of behavioral and biological factors was commonly mentioned:

*“Not all bodies have the same physique or strength. Some are more vulnerable, have less capacity, and it is also due to lack of care. We take from our body and in the end, we do not put in what it needs.” (Sugarcane worker, NIEHS)*

Health workers referenced genetic susceptibility as a risk factor for CKDnt, and, similar to labor leaders, were also concerned about other important environmental health influences. Both family members of the affected expressed a pre-determined view of CKDnt as reflected by this quote:

*“Even if you take care of yourself, the disease already comes in the body… in one’s genes. It is part of the family DNA.” (Wife of renal patient)*

### Understanding of informed consent

The vast majority (97%) of research participants remembered signing the consent form; however, over half (62%) did not recall any specific information on the document. While research participants broadly understood general concepts of genetics and disease, the lack of familiarity with genetic terms seemed to be a barrier to comprehension of informed consent. When asked to explain terms in the consent form (genetics/genética, genes/gen, DNA/ADN, genetic data/datos genéticos, and ancestry/ascendencia), only five research participants (14%) provided a response. The expressions typically used to define genes and DNA were blood type (*tipo de sangre)*, inheritance of characteristics and disease *(herencia de carácter y enfermedad)*, descendancy (*descendencia*), and ancestry *(ascendencia)*, as illustrated by these quotes:

*“Genes or genetics has to do with the collection of data that comes from your father, your father’s father… well, your descendants. The lineage from where we came.”(Sugarcane worker, NIEHS)*

*“DNA means that you look at the blood type of one and the blood type of the other, right?” (Former sugarcane worker, NIDDK)*

*“DNA is a disease, right? (Brick worker, NIDDK)*

*Genetic data are the DNA transferred from our parents and the possible characteristics or diseases that could be inherited from my generation to another generation or vice versa.”*

*(Miner, NIDDK)*

In the community stakeholder interviews, we provided examples of two types of consent: broad and specific. When we asked their opinions, health workers and labor leaders showed preference toward broad consent, because it was shorter and simpler. Other community members, mostly family members, preferred specific consent since they believed it would allow for the intake of information question by question, and would give the participant the opportunity to reflect. Health professionals were partial to broad consent, as it allowed for the use of information in future health studies.

Labor leaders and family members were unsure of what a ‘genetic study’ was and recommended that they also be informed of research studies, as they felt the benefits and risks apply to the whole community. Some community members thought researchers should review the consent form information with the participant’s family, while others, mainly health workers, suggested that family opinions might influence an individual’s personal decision.

### Attitudes towards data use and data sharing policy

When asked their opinions about the NIH data-sharing policy, nearly all research participants and community stakeholders required additional time, repetition, and/or explanation of the policies on the consent form, and often asked the interviewer to define “genetic data”. Fewer than half of the interviewees shared any opinion about the policy. However, the majority of the participants who did express opinions believed that more researchers with shared access to information meant more people able to contribute to solutions.

Participants who underwent the original consent form process had a vague comprehension of the fate of their biological samples. Most of these participants did not recall details about the usage of leftover samples for future research purposes, although they mostly noted that their samples would be stored in Nicaragua and later sent to investigators in the U.S. They were also more likely to mention that the original consenting process was rushed, and they would have liked an opportunity to weigh in on the opinions of loved ones.

Participants who underwent the supplemental consent process retained more information on details regarding the use and storage of samples, noting that the biological samples would be analyzed, and surplus samples would be stored in the Ministry of Health (MINSA) laboratories and later sent to the U.S. for future CKD-related research. They were also more likely to mention that the study data would be stored in a computer under a confidential de-identified code.

Many issues were observed with the comprehension of the consent form. The readability assessment on the original consent form (2,418 words) and the supplemental consent form (635 words) each generated a SOL score of 8.8, suggesting a ninth-grade education level is necessary for the comprehension of the text in the consent documents.

### Perceptions of benefits and risks of genetic research

Participants described several personal, community, and future benefits of research participation. For example, after blood and urine samples were collected and analyzed, the study protocol included the report-back of individual clinical results of kidney function parameters, such as serum creatinine and urinary protein, to participants during a consult with the study physician. Receiving lab results and consultations was often viewed by participants as health care they may otherwise not have access to:

*“The day they came to do my medical check- up, they gave me the results right then, for free. In other places, that amount of exams would not be free, I would get only what I could afford.” (Former sugarcane worker, NIDDK)*

Participants expressed understanding that the study may not lead to a cure for those currently affected by CKDnt, but they hoped that identifying the root cause of the disease might lead to a cure or prevention strategy for others in the future.

Only one participant raised concerns about the misuse or mismanagement of shared data and was worried about not knowing the intentions of the recipient:

*“Today in these times, you have to know how things are done. If these files are shared with others who you do not know, then other things can happen.” (Former sugarcane worker, NIDDK)*

Both groups of consented participants reported positive attitudes when asked about their experiences with the research team. They expressed gratitude for the information the team provided about CKDnt and how it helped them improve their health.

### Post-study expectations

Research participants expected the genetic component of the research to reveal whether the disease is inherited. Although most participants could not articulate why the genetic results from their samples were not returned, they had diverse expectations about the information genetic research would yield. Three participants believed the genetic study would identify defects in their genes, but only one of these participants also expected the researchers to inform the individual if they had a “bad gene”. The other two did not expect the researchers to return individual results because they believed the results were confidential and would be retained by research staff for in-depth investigation.

Several participants and community members, especially health professionals, expected updates that would communicate how the research was developing over time and sought information on partnerships with national health institutions:

*“Researchers need to give continuity, with a conclusion. We have to go into what this study arrived at, to see how the people are helped, through the same university or through the institutions that have to do with this, well in this case with MINSA, because it is a direct institution of the state for the care of patients.” (Health professional from León)*

The main results are summarized in Table 2.

**Table 2:** Summary of main results by domain of research.

Genetic research domains *n* (%)	Study participant *n* = 36	Community member *n* = 7
Views and knowledge		
Acknowledged associations between heredity and disease	34 (94)	6 (86)
Expressed deterministic view of disease onset	2 (1)	2 (29)
Expressed environmental view of disease onset	5 (14)	2 (29)
Believed behavioral and personal habits were causal factors	28 (78)	5 (71)
Consent form		
Remembered signing	35 (97)	-
Remembered information	14 (39)	-
Understood genetics terms on form	5 (14)	3 (43)
Provided opinion on NIH data-sharing policy	16 (45)	3 (43)
Perceived benefit	15 (42)	3 (43)
Perceived risk, concern about misuse of data	1 (<1)	0
Post-study expectations		
Expected researchers to identify genetic defects	3 (1)	0
Expected the return of individual results	1 (<1)	0

## Discussion

This study investigated research participant and community stakeholder perceptions of the informed consent process, policies, and expectations of research in order to recognize ethical concerns around genetic research conduct between high and low-income countries. Overall, our study highlights challenges with obtaining valid informed consent. The lack of familiarity with the terms presented in the consent form, amount of text, length of time for the consent process, and the number of times the consent form was addressed were underlying challenges precluding deeper discussion about biospecimens use, storage, and data-sharing policies.

The readability analysis suggests that a ninth-grade education level is necessary for the comprehension of the text in the consent document. Although there is currently no recommended reading level for health information aimed specifically toward Spanish-speaking populations, the NIH and American Medical Association (AMA) suggest a lower cut-off than the eighth-grade level [35]. Other health commissions recommend that materials should be written at or below a fifth-grade reading level to address the varying health literacy of all participants [37]. This raises the possibility that written individual consent forms that meet IRB guidelines may not be sufficient for relaying important elements of a research study to potential participants in LMICs [38].

Numerous studies indicate that consent procedures frequently fail to sufficiently inform participants with low incomes or low levels of literacy [39, 40]. It has also been noted that highly educated participants in high-income nations also struggle with understanding genetic research terms, concepts, and intent [41, 42]. Therefore, although literacy and education levels are important factors in the comprehension of information, understanding the genetic research process is also dependent on the efforts and methods researchers use to provide the information [42, 43]. Multiple studies propose alternate models of informed consent that go beyond text and incorporate visuals, like timelines, explanatory imagery, and storytelling into the explanations of the study [13, 19, 44]. Our findings suggest that it may also be helpful to review the study aims and policies in the consent form with the participant more than once; when results are returned and during any other follow-ups.

Participants in both high and low-income settings have reported difficulties with distinguishing research concepts from health care [14, 44]. Although no personal health benefits to research participation were listed in the consent form, nearly all study participants perceived the return of individual results as a clinical benefit, as they received information relevant to understanding their personal health; however, the information may not be clinically actionable in LMIC settings. This perception may lead to expectations around the responsibility of researchers to provide necessary, or ancillary, care to kidney patients [45]. To help set realistic expectations of research, the consent form could have illustrated how genetic research concepts, technologies, and capabilities differ from primary and specialty care.

Our participants and community stakeholders expected the research to yield individual and social benefits but seldom alluded to potential risks and harms, likely because there is not much history with genetic research in Central America. There is, however, a long history of misconduct and lapses in research ethics among Indigenous communities [46]. The genome of Latin American populations reveals haplotypes associated with Indigenous, African, and European groups [47]. When using, sharing, and discussing the genetic data of those with Indigenous ancestry (who are not protected by tribal regulations), there should be careful consideration of unintended consequences, as findings may generate assumptions about the genetic susceptibility of other populations who descend from Indigenous groups in the Americas. Statements about the genetic vulnerabilities of families and communities may lead to discrimination, social stigma, and limited economic opportunities. These realities have informed the framework for the conduct of ethical genetic research with Indigenous groups, and serve as a foundation for researchers conducting studies with diverse communities in Central America [46, 48].

Studies that have explored stakeholder perspectives of genetic research in LMICs and Indigenous communities highlight capacity building as a potential solution to some of the challenges with comprehension of genetic study procedures [48, 49]. The development of long-lasting relationships between ethics committees, research committees, health agencies, geneticists, nephrologists, epidemiologists, community leaders, study participants, health workers, private groups, and other stakeholders promotes the ethical practice of genetic research. These relationships facilitate conversations about culturally appropriate ways to disseminate information on genetic studies and findings. Additionally, there is a need to strengthen the capacity of health agencies in LMICs to access, analyze, and interpret publically available genomic data through improvements in technology and training [50].

There are a few limitations to this study that are noted. We intended to facilitate focus group interviews; however, this was unrealistic as the study took place during a time when strict COVID precautions were in place. Participants had similar initial reactions to the NIH policy questions during the individual interviews. Focus groups could have generated clarifying questions, leading to more dialog. Another limitation is that we did not objectively measure genetic literacy using any known measures, and thus are not able to analyze whether attitudes/concerns varied as a function of genetic literacy. In addition, different languages were used during data collection and for data analysis, which creates a potential for meanings to be lost or misconstrued during translation. Finally, we did not evaluate differences between cases and controls.

## Conclusion

Little to no genetic research has been conducted in Nicaragua. We sought to understand the views of participants and community members acquainted with current studies to inform future efforts. We found that the informed consent document is not well understood among participants and community members in Nicaragua, raising ethical concerns in the conduct of genetic research in this setting. We identified three areas of research to improve upon: (1) information delivery and the informed consent process, (2) differentiation between research and medical care (3) discussion of potential ELSI of genetic research findings. Building capacity is a potential solution to these challenges, as strengthening relationships facilitates communication and engagement with insider experts.

## Supplementary information

APPENDIX

## Data Availability

The datasets generated and/or analyzed during the current study are available from the corresponding author on reasonable request.
