# Supplementary material for: Ethical considerations for genetic research in low-income countries: perceptions of informed consent, data sharing, and expectations in Nicaragua
Source: Eur J Hum Genet. 2023 Dec 5;32(10):1278–84. doi: 10.1038/s41431-023-01505-7 (PMC11500004; doi:10.1038/s41431-023-01505-7)
Supplement: Supplementary file 1 — APPENDIX [file 41431_2023_1505_MOESM1_ESM.docx]

*
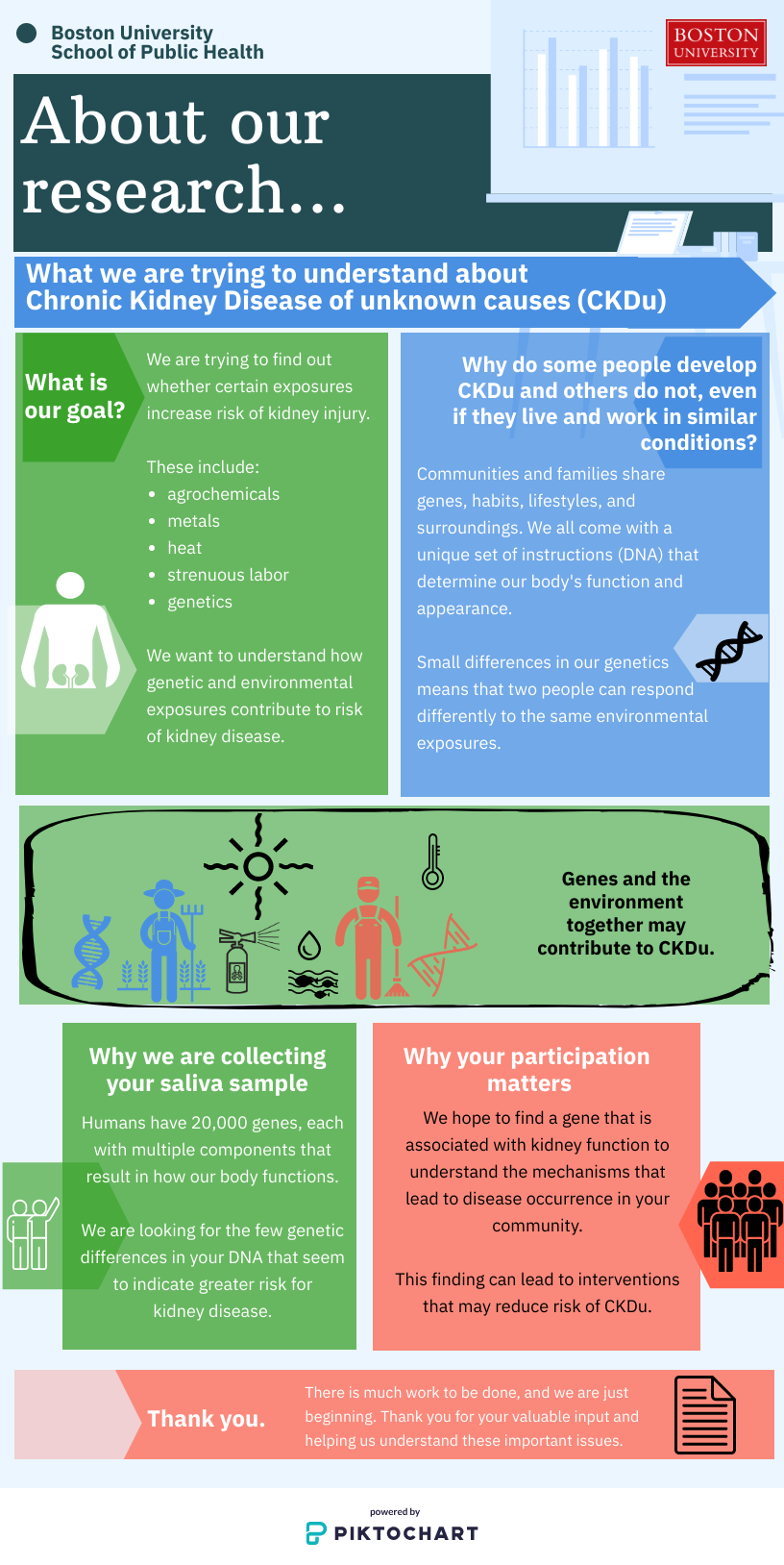
***APPENDIX A. Post-Interview Infographic**

**APPENDIX B. SMOG formula and SOL conversion formula**

The SOL scale, also referred to as the Spanish SMOG (Simple Measure of Gobbledygook), is an adaptation of the SMOG index, a method that is widely used to determine how easy or difficult English text is to read and comprehend by counting the number of polysyllabic words and sentence lengths [33-35]. The figures below show the SMOG formula and the corresponding SOL conversion equations.

*
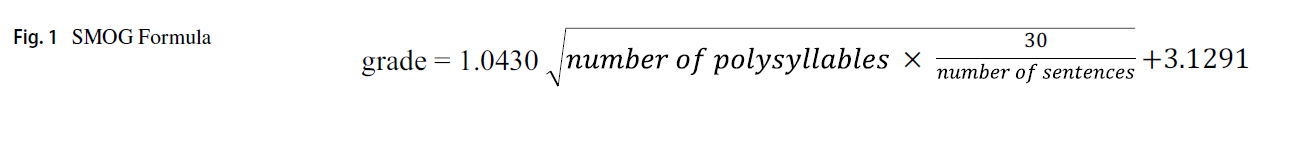
*

*
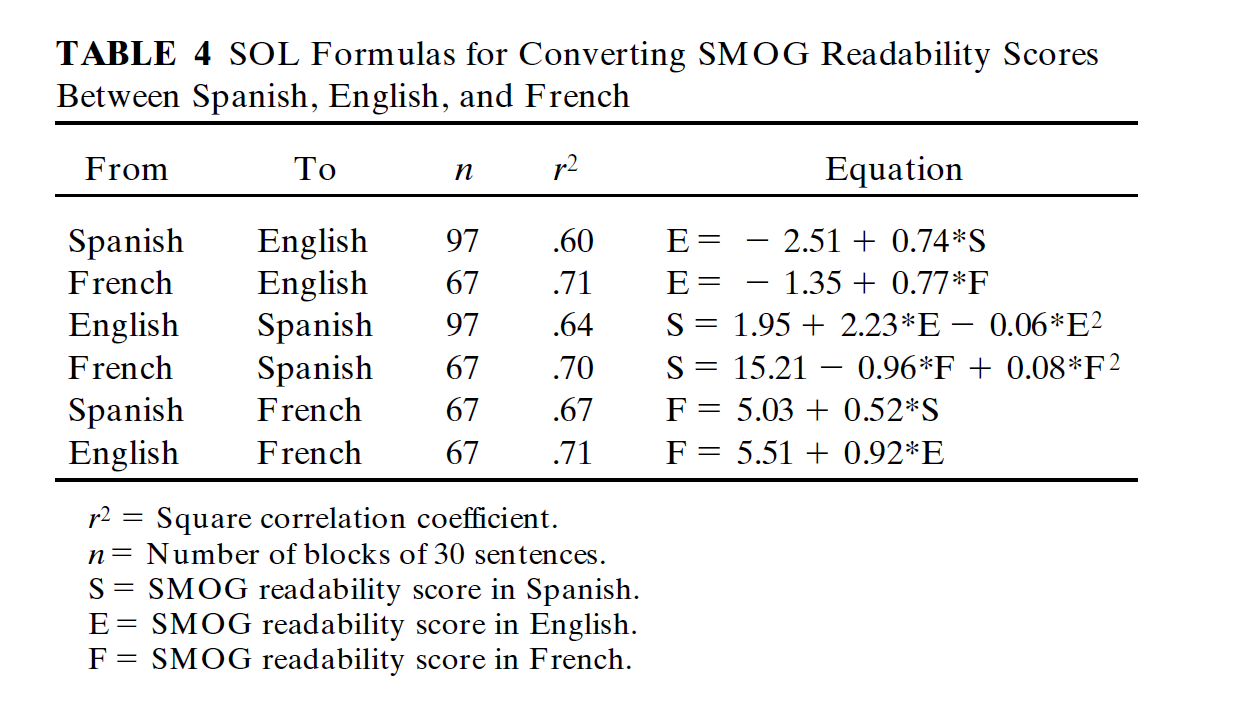
*
